# Gelatinase (MMP-2 and -9) expression profiles during gestation in the bovine endometrium

**DOI:** 10.1186/1477-7827-6-66

**Published:** 2008-12-31

**Authors:** Keiichiro Kizaki, Koichi Ushizawa, Toru Takahashi, Osamu Yamada, Junichi Todoroki, Takashi Sato, Akira Ito, Kazuyoshi Hashizume

**Affiliations:** 1 Laboratory of Veterinary Physiology, Department of Veterinary Medicine, Iwate University, Ueda 3-18-8, Morioka, Iwate 020-8550, Japan; 2 Department of Developmental Biology, National Institute of Agrobiological Sciences, Ikenodai 2, Tsukuba, Ibaraki 305-8602, Japan; 3 Miyagi Prefectural Animal Health Hygiene Ogawara Station, Miyagi 989-1243, Japan; 4 Team Todoroki ARR, Kurahara, Miyakonojo, Miyazaki 855-0051, Japan; 5 Department of Biochemistry and Molecular Biology, Tokyo University of Pharmacy and Life Science, Hachioji, Tokyo 192-0392, Japan

## Abstract

**Background:**

Various molecules participate in implantation and maintaining endometrial function during gestation. The remodeling of endometrial matrices is a necessary process in the coordination of gestational progress. Matrix-metalloproteinases (MMPs) like gelatinases (MMP-2 and -9) and collagenase (MMP-1) are considered to play important roles in this process. We examined MMP-2 and -9 expression using zymography, in situ hybridization, real-time PCR, and microarray analysis to clarify their roles in the bovine endometrium during gestation.

**Methods:**

Endometria, placentomes, and fetal membranes were collected from Japanese black cows that were killed on day 15 to 252 of gestation or during their estrous cycle. The gene expression of MMP-related molecules (mainly MMP-2 and -9) was examined using a custom-made microarray, real-time RT-PCR, and in-situ hybridization. Gelatinase activity was detected by zymography and film in situ zymography.

**Results:**

Both gelatinases were expressed in the endometrium and fetal tissues throughout gestation. MMP-2 gene expression declined with the progress of gestation, but its intensity was maintained at a high level during the peri-implantation period and increased in late gestation. The expression level of MMP-9 was stably maintained, but was relatively low compared to that of MMP-2. These gene expression patterns matched those detected by zymography for the proteins. Microarray analysis suggested that the functions of MMP-2 during implantation and the last part of gestation are closely related with those of other molecules such as tissue inhibitors of metalloproteinase (TIMP)-2, a disintegrin and metalloproteinase with thrombospondin motifs (ADAMTS) 1, membrane type 1 (MT1)-MMP, and extracellular matrix metalloproteinase inducer (EMMPRIN).

**Conclusion:**

We detected MMP-2 and -9 gene expression in the bovine endometrium and placentome throughout gestation. These data suggest that MMP-2 is one of the main endometrial remodeling factors for implantation and pre-partum in cattle. In cows, as is the case in humans and rodents, gelatinases participate in endometrial remodeling, and their activities depend on the balance of activators and inhibitors; i.e., TIMP, MT-MMP, EMMPRIN, MMP-2, MMP-9, and so on.

## Background

Implantation is a complex process and involves various factors such as cytokines and hormones. Degradation and regeneration of the endometrial extra-cellular matrix (ECM) is a vital process that involves matrix metalloproteinases (MMPs). MMP, including MMP-2 (gelatinase A) and -9 (gelatinase B), play an important role in tissue remodeling in various physiological and pathological processes, such as implantation, ovarian and uterine functions during peri-partum, wound healing, cancer development etc. [1,2]. Although the roles of MMP during implantation are known for invasive placentation as occurs in humans and rodents [3-9], knowledge concerning endometrium remodeling in non-invasive placentation, as occurs in ungulates, is limited. The important roles of MMP in endometrial remodeling, especially those of MMP-1 and -2, were reported in sheep [10,11]. Interstitial cells are responsible for their production, but epithelial cells are not present in the ovine endometrium [10]. In goats, MMP-2 activity is regulated by co-localized membrane-type 1 MMP (MT1-MMP) and tissue inhibitor of metalloproteinase-2 (TIMP-2), and they control endometrium remodeling during gestation [12]. In cattle, trophoblast cells attach to the surface of the epithelium on the caruncle, which is a specific region for implantation, and then the trophoblast cells and endometrial epithelia fuse and make binucleate or multinucleate cells on the caruncle [3,13,14]. Although, the roles of MMP have been reported during peri-partum, termination of gestation, and post partum in cows [2,15], the detailed expression profiles of gelatinases have not been clarified during implantation; namely, the proteolysis mechanisms of the endometrial ECM are still obscure during implantation in cows. Many species of MMP and related molecules participate in this process, including TIMP and MT-MMP [1]. During the implantation process, trophoblast cells eliminate epithelial cells, and the epithelium is reorganized [13,16]. Gelatinases may play a significant role in this process. After implantation, the newly formed binucleate and multinucleate cells produce many molecules, peptides, and hormones, such as placental lactogen, pregnancy-associated glycoproteins, prolactin-related proteins, steroid hormones, and heparanase [3,13,17,18]. Compared to invasive placentation, the mechanisms of this type of proteinase is unknown in cows, but it is just as important. Most embryo loss occurs during the early implantation period in cows. The cause of this usually relates to the uterine circumstances, which depend on humoral regulation and may depend on the spacio-temporal condition of the endometrium. Abnormalities in endometrial function cause early embryo loss in many species, and MMPs participate in the regulation of endometrial functions in ruminants [11,19,20]. In the present study, MMP-2 and -9 mRNA were analyzed quantitatively using real-time PCR and microarray analysis, and the expression of their proteins was confirmed by gelatin zymography.

## Methods

### Animals and tissue collection

Japanese black cows were killed on day 15 to 252 of gestation or during their estrous cycle, and placentomes, endometria, and fetal membranes were collected. Pregnancies were derived by artificial insemination (AI) (day 0 was the day of insemination). Detailed tissue collection data are shown in Table 1. Caruncular (CAR), intercaruncular (ICAR), cotyledonay (COT), and intercotyledonary (ICOT) tissues were collected separately for mRNA extraction and zymography. The tissues collection process was as follows: The caruncle area was cut out together with a small area of the intercaruncular endometrium because of the difficulty of complete mechanical separation of the caruncle and intercaruncular tissue. Therefore, the caruncle tissue samples contained a small amount of intercaruncular tissue. The placentome was mechanically divided into the mother side (caruncular septa) and fetal side (cotyledonay villi) tissues; however, they contained a small amount of tissues from the opposite side. On days 15 to 30 of gestation, the cotyledon on the fetal membrane could not be easily identified so the cotyledon samples contained both tissues. A portion of the endometrium and fetal membrane was collected and embedded in OCT compound (Tissue-Tek OCT compound, Sakura Finetechnical Co., Ltd. Tokyo, Japan) for *in situ *zymography. These tissues were frozen in liquid nitrogen and stored at -80°C until sectioning. Some uterine samples were perfused with 4% (w/v) paraformaldehyde in 10 mmol PBS l^-1 ^(pH 7.4) via the ovarian and uterine arteries. After perfusion, the uteri were separated into non-gravid and gravid horns. Each horn was sliced into sections of about 10 mm thickness and post-fixed in 4% (w/v) paraformaldehyde in PBS overnight at 4°C. The post-fixed tissues were dehydrated in alcohol and xylene, and embedded in paraffin wax. The mounted samples were cut into 5–7 μm sections with a rotary microtome HM355 (MICROM Laborgeräe GmbH, Heidelberg) and placed onto slides (Matsunami, Tokyo) for *in situ *hybridization analysis.

**Table 1:** Tissue collection schedule.

Symbol of group	Actual collection days	No. of animals	Definition for purposes
Estrous cycle			
E0	day 0	2	QPCR, in situ hybridization
E7	day 7	2	QPCR
E13	day 13	2	microarray
			
Gestation			
Day 15	day 15*,19, 21*	3	QPCR, *zymography (*in situ*)
Day 21	day 21	1	*in situ *hybridization
Day 25	day 25	2	microarray
	day 25, 32	2	zymography (extraction)
Day 30	day 27, 30, 34	3	QPCR
Day 60	day 56, 56, 60, 64	4	QPCR, microarray
Day 150	day 97, 148, 150	3	QPCR, microarray
	day 144, 148,150	3	zymography (extraction)
Day 250	day 245, 249, 252	3	QPCR, microarray, zymography (extraction)

QPCR: Real-time RT-PCR. *: Each of them was used for *in situ *zymography as shown Day 15 and Day 21 in Figure 4.

All other tissue samples were immediately frozen in liquid nitrogen and stored at -80°C until mRNA and protein extraction. They were then used for microarray, real time PCR, and zymography analysis. Before day 21 of gestation, the achievement of gestation was confirmed by flushing the embryo from the uterus. After day 21, pregnancy was confirmed by transrectal ultrasonography (7.5 MHz linear probe, SSD-1700 Aloka, Tokyo, Japan). The viability of the fetus between days 25 and 60 of gestation was confirmed by a detectable heartbeat 2–3 days prior to slaughter.

### cDNA microarray

We used a custom-made utero-placental cDNA microarray that was developed in our laboratory as previously described [21,22]. In brief, a cDNA library was constructed from mRNA isolated from endometrial (caruncular and intercaruncular endometrium) and placental tissues (cotyledonary and intercotyledonary fetal membrane) of Japanese black cows. The PCR products of about 4,000 clones from the cDNA library were robotically spotted onto glass slides. The clones were simultaneously sequenced using the MegaBACE 1000 DNA Sequencing System (Amersham Pharmacia Biotech, Piscataway, NJ). The array contained 3,955 spots that were clustered into 1,738 unique genes on the basis of sequence analysis. An additional 35 genes that were not included in the cDNA library but had also been spotted onto the cDNA microarray were used for the analysis since these genes have been shown to be characteristically expressed during gestation in bovines and humans [4,11,19,23-27]. MMP related genes made up the bulk of this group. The cDNA microarray hybridization procedures were described in previous reports [21,22]. Briefly, two μg of poly (A)+ RNA were reverse transcribed in the presence of cyanine 3 (Cy3) or Cy5 fluorescence dye (Amersham Biosciences, Buckinghamshire, UK) using SuperScript II reverse transcriptase (Invitrogen) to make the hybridization probes. Identical samples were labeled separately with either Cy3- or Cy5-dye. Thus, two hybridization reactions could be carried out with the same sample. The arrays were sequentially washed with different concentrations of SSC solutions after 16 hr incubation at 65°C. The arrays were dried by centrifugation at 1,000 × g. Hybridization images were immediately scanned by a GenePix 4000B laser scanner (Axon Instrument, Union City, CA, USA) and analyzed with GenePix Pro 4.0 software. Data normalization was performed by previously described procedures [26,27]. The local background intensity of each array spot was smoothed by local weight regression (Lowess) and subtracted from the spot intensity data. The subtracted intensity data were subjected to non-parametric regression and local variance normalization since non-parametric regression can reduce intensity-dependent biases. The accuracy is improved over that of linear regression if the points in the scatter plot of Cy3 versus (vs.) Cy5 are not distributed around a straight line. Data for individual genes were obtained by averaging the intensity values of analogous spots on the microarray. Data were log_2 _transformed and used for cluster analysis. The Cluster 3.0 program was used for the hierarchical clustering. The hierarchically clustered data were visualized using the TreeView 0.99 program (M.B. Eisen's based clustering program [28], .

### Real time RT-PCR

The levels of MMP-2 and -9 gene expression were analyzed with real time RT-PCR using a Taqman probe as described previously [29]. Real time PCR primers were designed using sequence data. Partial bovine MMP-2 and -9 mRNA strands were amplified and identified as follows: Total RNA was isolated using ISOGEN (Nippon Gene, Kyoto, Japan), and one microgram of total RNA was subjected to reverse transcription of cDNA with transcriptase (Invitrogen, Carlsbad, CA, USA) according to the manufacturer's instructions. After transcription, 5 μl were used for the PCR amplification with DNA polymerase (Boehringer Mannheim). The PCR fragments were subcloned into a TA vector (Invitrogen). Both strands were sequenced with a DNA sequence kit using a sequencer (prism 377 DNA Sequencer ABI). We used oligonucleotide primers for cDNA cloning and quantitative real-time RT-PCR analysis are listed in Tables 2 and 3. After confirming their sequence identification, their fragments were used as the standard for measuring their relative expression. All other reagents for mRNA analysis were purchased from Sigma-Aldrich Co. (Saint Louis, MI, USA) or Wako Pure Chemical Industries, Ltd. (Tokyo, Japan).

**Table 2:** Oligonucleotide primers used for cDNA cloning

Gene	Primer	Sequence	Position
MMP-2	Forward	CCACGTGACAAGCCCATGGGGCCCC	1–25
(AB043994)	Reverse	GCCAGCTCAGCAGCCTAGCCAGTCG	489–471
			
MMP-9	Forward	GTGTACACCGGCGCGTCG	1786–1803
(X78324)	Reverse	GGGCACTTCAGGAGGTCG	2135–2118
			
GAPDH	Forward	CCTTCATTGACCTTCACTACATGGTCTA	173–200
(NM_001034034)	Reverse	GCTGTAGCCAAATTCATTGTCGTTACCA	1029–1003

**Table 3:** Oligonucleotide primers used for quantitative real-time RT-PCR analysis

Gene	Primer	Sequence	Position
MMP-2	Forward	AAAATGGATCCTGGCTTCCC	295–314
(AB043994)	Reverse	AATAGGCGCCCTTGAAGAAGT	415–395
	Probe	TGCCTGGAACGCCATCCCTGATAA	330–353
			
MMP-9	Forward	TCGACGTGAAGACACAGAAGGT	1925–1946
(X78324)	Reverse	TGATCCTGGCAGAAGTAAGCTTTC	2051–2028
	Probe	CATTAGCACGCACGACATCTTTCAGTACCA	1995–2024
			
GAPDH	Forward	AAGGCCATCACCATCTT	280–296
(NM_001034034)	Reverse	CCACTACATACTCAGCACCAGCAT	355–332
	Probe	AGCGAGATCCTGCCAACATCAAGTGG	302–327

### Measurement of MMP-2 and MMP-9 activity by gelatin zymography

About 150 – 400 mg of endometrial tissues including placentomes from day 25 to 252 of gestation (shown in Table 1) were placed in Tris-buffer saline (50 mM Tris ph 7.5, 150 mM NaCl, 1 mM CaCl2, 0.05% Briji 35, 10 μg/ml Leupeptin, 1 mM PMSF) and then homogenized with Polytron (Kinematica, Swizerland). The homogenates were centrifuged at 20,000 × g, and the collected supernatants were used for gelatin-zymographic analysis.

The MMP-2 and -9 activity was detected by gelatin zymography as described previously [30,31]. Briefly, 10 μg protein in 10 μl Tris-buffer were loaded onto each gelatin-containing lane for SDS PAGE (12.5% SDS gel contained 1 mg/ml gelatin) and were separated using mini-gel apparatus (Bio-Rad, NY, USA) under non-reducing conditions. After electrophoretic separation, the gels were washed at least three times for 10 min with extraction buffer (50 mM Tris-HCl, 5 mM CaCl_2_, 1 μM ZnCl_2_, and 0.02% (w/v) NaN_3_, pH7.5, containing 2.5% (w/v) Triton X-100) at room temperature and incubated in incubation buffer (same composition as the extraction buffer but without 2.5% Triton-X100) for 16 hr at 37 C. The gel was stained with Coomassie Brilliant Blue R-250 in 30% (v/v) methanol and 10% (v/v) acetic acid in H_2_O. After washing away the excess dye with distained solution (45% (v/v) methanol and 10% (v/v) acetic acid), the gelatinolytic activities of MMP-2 and -9 were analyzed with NIH imager 1.62. The proteolytic activity appeared as clear bands on a blue background. The relative molecular mass (Mr) of gelatinolytic MMP were determined by comparison with SDS-PAGE molecular weight protein markers (Bio-Rad) in the adjacent lane. In order to determine the type of gelatinase observed on the zymograms, in one experiment, 10 mM ethlenediaminetetraacetic acid (EDTA; Ca^2+ ^chelator) was added to the buffer during the incubation period. All procedures were performed as they were for gels without EDTA in the incubation buffer. Data are shown as arbitrary units by NIH image after adjusting the background intensity and the relative units to represent the latent form of MMP-2 on Day 0 of the estrous cycle as 1.

### *In situ *hybridization

The full-length bMMP-2 (accession no. AB043994) and bMMP-9 (accession no. X78324) cDNAs were used as templates for hybridization probe synthesis. Digoxigenin (DIG)-labeled antisense and sense-complementary RNA probes were prepared as described in previous studies [32,33]. The placentomes were sectioned into 7 μm-thick sections. *In situ *hybridization was performed using the automated Ventana HX System Discovery with a RiboMapKit and BlueMapKit (Ventana, Tucson, AZ, USA) [32,33]. Briefly, bovine placentomal sections were hybridized with DIG-labeled probes in RiboHybe (Ventana) hybridization solution at 67°C for 6 hours. The sections were washed three times in RiboWash (Ventana) (67°C, 6 min) after hybridization and fixed in RiboFix (Ventana) (both 37°C, 10 min). The hybridization signals were then detected using a monoclonal-anti-digoxin biotin conjugate (Sigma). The hybridized slides were observed after preparation with a Leica DMRE HC microscope (Leica Microsystems, Wetzlar, Germany) with a Fujix digital camera HC2500 (Fujifilm, Tokyo, Japan).

### Film *in situ *zymography

Localization of endometrial gelatinase activity was detected by film *in situ *zymography according to the method of previous reports [34,35]. We used GN film (Fuji Photo Film Co., Ltd, Tokyo, Japan) coated with a gelatin base emulsion to detect and localize the gelatinase activity in the underlying tissue. Seven-micron cryostat unfixed endometrium sections were mounted onto the coated film, followed by incubation for 12 h at 37°C, and staining with bearish scarlet (BS; Chroma-Gesellschaft, mbH & Co., Munster, Germany) and hematoxylin. Gelatinase activity was detected when a pattern of gelatin digestion was found that was in accordance with gelatinase localization as recorded by a white color caused by the weaker staining of BS.

### Statistics

Data were analyzed initially by ANOVA and then by a Turkey-Kramer multiple comparison test. P-values of < 0.05 were considered significant.

## Results

### Microarray analysis of MMPs related genes

**Figure 1:**
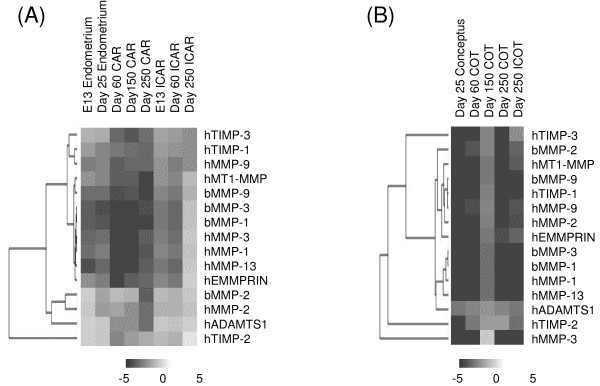
**Hierarchical clustering of MMP and related gene expression during gestation**. The black cells indicate down-regulation, and the white cells indicate up-regulation. Gray cells indicate no change in expression. A: endometrium, B: fetal tissues. Two animals were used on each day, and the array was done twice and the data shown represent the mean value. The data show a representative day as in Table 1. CAR: caruncule, ICAR: intercaruncle, ICOT: intercotyledon, COT: cotyledon. Microarray spots cDNA were created using the following genetic information: bovine MMP-1 (AF134714), bovine MMP-2 (AB043994), bovine MMP-3 (AF135232), bovine MMP-9 (X78324), human EMMPRIN (L10240), human MMP-1 (NM_002421), human MMP-2 (J03210), human MMP-3 (NM_002422), human MMP-9 (NM_004994), human MMP-13 (NM_002427), human MT1-MMP (NM_004995), human TIMP-1 (NM_004995), human TIMP-2 (J05593), human TIMP-3 (U14394), and human ADAMTS1 (AF207664).

Various MMPs related genes were analyzed with a custom-made utero-placental cDNA microarray as shown in Figures 1A and 1B. In ICAR, many genes including MMP-2, MMP-9, MMP-1, MMP-3, TIMP-2, MT1-MMP, extracellular matrix metalloproteinase inducer (EMMPRIN), and a disintegrin and metalloproteinase with thrombospondin motifs (ADAMTS) 1, showed higher expression in late gestation compared to other gestation periods. These expression intensities were stronger than those in CAR. The intensity of MMP-2 expression was stronger than those of other MMP-related genes throughout gestation. MMP-2 was distributed in a different compartment from MMP-9; however, ADAMTS1 and TIMP-2 occupied compartments close to MMP-2. In the fetal side, the intensities of most genes were rather weak compared to those in the endometrium, but in the middle of gestation the activity of all the genes was increased compared to other periods of gestation. Hierarchical clustering showed that MMP-2 and MMP-9 were distributed in the same compartment in fetal tissues.

### Gelatinase mRNA expression profiles obtained with quantitative PCR during gestation

**Figure 2:**
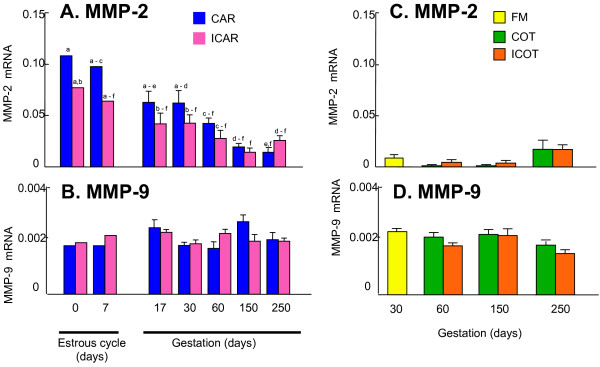
**Gelatinase mRNA expression during gestation as analyzed by quantitative PCR**. The expression values were normalized with cloned MMP-2 and MMP-9 as standards. Expression refers to the mean +/- SD. Two cows were used on each day during the estrous cycle, and three cows were used on each day during gestation. The abbreviations are the same as in Figure 1. A: MMP-2 in endometrium. B: MMP-9 in endometrium. C: MMP-2 in fetal side tissues. D: MMP-9 in fetal side tissues. a-c:abc; a-d: a, b, c, d; a-e: a, b, c, d, e; a-f: a, b, c, d, e, f; b-f: b, c, d, e, f; c-f: c, d, e, f; d-f: d, e, f. Different letters above each bar indicate significant difference at P < 0.05. No significant difference was found in Figures 2B, 2C and 2D.

The microarray data suggest that MMP-2 has an initiating role in endometrial remodeling throughout gestation. Therefore, we focused on gelatinase expression and analyzed the profiles in more detail using quantitative RT-PCR (Fig. 2). The highest MMP-2 expression was found during the estrous cycle, although rather high expression remained in early gestation. During the middle of gestation, MMP-2 expression was decreased and fell to low levels with as gestation progressed. The level was 3- to 4-times less than that observed during the estrous cycle. The expression levels in CAR were higher than those in ICAR during the estrous cycle and early gestation. There were no significant differences between the levels in the intercotyledon and cotyledon. In cotyledonay tissue, MMP-2 expression was lower than in the endometrium throughout gestation.

MMP-9 expression in the endometrium remained stable throughout the estrous cycle and gestation, but rather low values were detected compared to those of MMP-2. There were no significant differences between the levels of CAR and ICAR. The MMP-9 expression levels in the fetal side tissues including the fetal membrane, cotyledon, and intercotyledon were stable throughout gestation. The expression levels were similar to those of the endometrium.

### Gelatinase production in the endometrium

**Figure 3:**
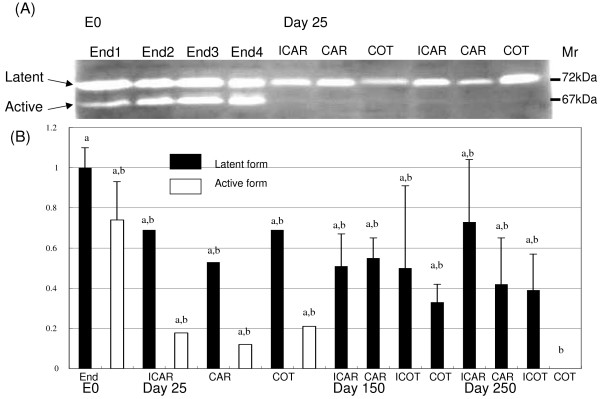
**Gelatinase activity in the bovine endometrium during gestation according to zymography**. A: Representative zymography gel data. B: Gelatinase activity in the placentome and fetal tissues. The data show the latent form activity as 1 in the endometrium on Day 0 of the estrous cycle (E0). The latent form MMP-2 is about 72 kDa in size and the active form is about 67 kDa. Triplicate repeat analyses were performed for each sample and the mean +/- SEM is shown after being analyzed by NIH image as outlined in the method section of the text. The tissue sample numbers is shown in Table 1. Different letters above each bar indicate significant difference at P < 0.05.

Endometrial MMP-2 production was stable during the estrous cycle and early gestation; however, the active form of MMP-2 decreased markedly in the endometrium on day 25 of gestation as shown in Figures 3A and 3B. Both the latent and active forms maintained lower production during middle and late gestation not only in the maternal tissues; e.g., the caruncular and intercaruncular endometrium, but also in the fetal tissues, such as the cotyledon and fetal membrane. The expression of the active form was much lower than that of the latent form; the active forms of ICAR, CAR and COT in the endometrium on Day 25 of gestation showed relative expression levels of 0.74, 0.17, 0.12, and 0.21, respectively, where 1.0 represents the latent form in the endometrium on Day 0 of the estrous cycle. Almost none of the latent form was detected throughout gestation. Although MMP-9 production also decreased as gestation progressed, it was much lower compared to that of MMP-2, and no visible expression was detected by gelatin zymography during gestation.

### *In situ *zymography and *in situ *hybridization

**Figure 4:**
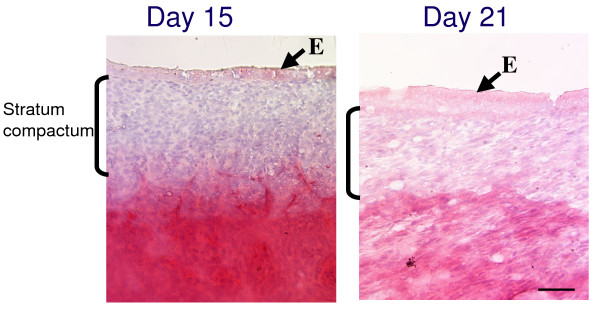
**Detection of gelatinase activity in the endometrium during the pre-implantation period according to *in situ *zymography**. Day: day of gestation, E: epithelium. Clear parts or less staining with biebrich scarlet indicate gelatinase activity. Bar = 50 μm.

Conventional zymography showed that MMP-2 expression surged during the peri-implantation period in bovines; however, its gene expression decreased with the progression of gestation. The activity of gelatinase was confirmed using endometrial tissues from around the time of implantation by *in situ *zymography. The data showed that the activity on Day 21 was stronger than that on Day 15 of gestation as shown in Figure 4. The activity was detected in all endometrial parts including the epithelium and stroma. On Day 15, the activity was limited to the stratum compactum, but the activity eventually spread out over the whole endometrium.

**Figure 5:**
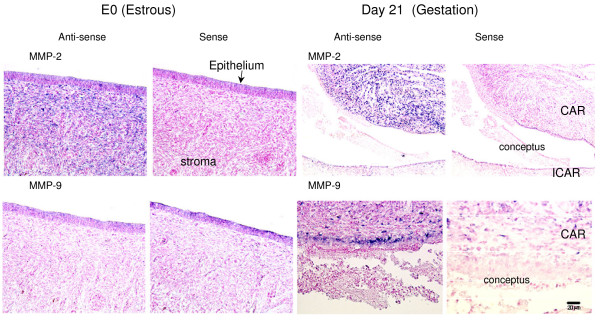
**Gelatinase mRNA expression in the endometrium during the peri-implantation period**. CAR: caruncular endometrium. ICAR: intercaruncular endometrium. Bar = 20 μm.

The *in situ *hybridization data of both gelatinases (Fig. 5A) supported that of the *in situ *zymography. MMP-2 expression was found in the stroma on Day 0 of the estrous cycle, but MMP-9 showed no signal in the endometrium. On Day 21 of gestation, an intense MMP-2 signal was noted for CAR, and an MMP-9 signal appeared in the stratum compactum. The expression of both genes was detected at the feto-maternal interface in CAR.

## Discussion

In this study, we found that gelatinases were expressed in the endometrium throughout gestation and that MMP-2 expression surges during the peri-implantation period. We propose that MMP-2 plays a vital role in the cell to cell contact and remodeling of endometrial epithelia and stoma during the implantation period in cows. To support this hypothesis, we gathered various pieces of evidence. First, zymography showed that the MMP-2 activity in the endometrium is highest during the preimplantation period. Second, *in situ *zymography showed that endometrial epithelia and stroma express gelatinase activity during implantation. Third, quantitative PCR revealed that the level of endometrial MMP-2 expression is more than ten-times greater than that of MMP-9. Fourth, microarray data confirmed that MMP-2 expression is higher than those of MMP-9 and other related proteins.

Endometrial remodeling is essential for successful implantation, and MMP have been shown to regulate this process in various species [4,7,11,36,37]. MMP are proteolytic enzymes that depend on zinc and calcium ions, and are the main degradation factors for the ECM in various tissues [1]. In non-invasive placenta species, our knowledge of the roles of MMP is limited. In bovines and sheep, the importance of gelatinase activities for placental formation and fetal growth have been reported throughout gestation, and their role in labor was confirmed [14,15]. A previous report showed no marked expression of MMP-2 or -9 proteins in the bovine placenta during late gestation, but MMP-2 appeared during labor [14]. Specifically, MMP-2 function seems to be a key factor for the release of the placenta in cows [38]. These expression patterns match those found in the present study. This suggests that endometrial gelatinases remain in ruminants during the later half of gestation, and MMP-2 activity recurs around labor. This appearance may be a necessary event for placental release after labor in ruminants, although collagenase plays the main role in the separation of the placenta [39,40]. Retention of the fetal membrane is found in bovines as a major post partum problem and collagenase treatment accelerates the release of the fetal membrane. The mechanism of regulation of this process depends on placental cell function and endocrine factors [41,42], which may affect MMP-2 expression and regulation.

MMP-2 and MMP-9 activity declined in the caruncular area and cotyledon during late gestation but were slightly increased in the inter-caruncular area and inter-cotyledon. This suggests that gelatinases are required for fetal membrane expansion during fetal growth, but placental matrices have to be maintained until delivery to maintain gestation. TIMP-2 is an inhibitory regulation molecule for MMP-2 that is strongly expressed in the endometrium during late gestation, and it may be necessary for maintaining a balance between degradation molecules and their inhibitors.

Microarray analysis is a useful tool for elucidating the complex expression patterns of many genes simultaneously [43,44]. In fact, we examined various MMP related genes with a custom arranged bovine utero-placental cDNA array [21]. The data obtained suggest that the expression of various MMP-related molecules is scarce but that they each have specific activities that degrade the ECM. Their activities may have a mutual activation process in endometrial degradation. The microarray analysis suggested that ADAMTS1, TIMP-2, and EMMPRIN are involved in MMP-2 expression, but their regulatory mechanisms remain to be studied. In the bovine endometrium, MMP-2 may mediate ECM degradation [16,18,38] since its protein production and gene expression were maintained during the implantation period. Then, its appearance decreased with the progression of gestation. ECM including collagen type-I, -IV, laminin, and fibronectin expression declined around the start of implantation both spatially and temporally [16]. This is compatible with findings from rodents, humans, and even bovines with a non-invasive placenta [6,8,45]. Therefore, MMP-2 may play significant regulatory roles for gelatin and collagen type IV during implantation, even in ruminants. In the current study, MMP-2 expression was lower during the implantation period compared to during the estrous cycle but it still maintained a high level. Zymography showed that the expression intensity of MMP-2 in the endometrium and trophoblasts was higher that of MMP-9 during the peri-implantation period and other gestational periods. These results preliminarily accorded with the microarray and real-time PCR analysis but did not completely match. As we found faint gelatinase activity in the endometrium and fetal tissues, there are discrepancies between the transcription and translation of these enzymes. *In situ *zymography data also suggests that the activation of these enzymes does not depend on the intensity of gene expression and also that activation processes are more important for these enzymes because they are produced from zymogenes. *In situ *hybridization and *in situ *zymography data indicate that the modified enzyme is not required in all areas of the endometrium. Around implantation, trophoblast cells displayed high MMP-2 intensity, and after implantation less than half of MMP-2 was detected in the caruncle. Endometrial stroma cells may produce less MMP-2 around implantation [9], and stromal MMP-2 production is regulated by trophoblasts [46]. The cell-to-cell interaction depends on the balance of MMP production and inhibition by MT-MMP, TIMP, and EMMPRIN [47,48]. Spatial cell interaction at the feto-maternal interface occurs, and specific molecules of fetal origin participate in endometrial remodeling, and trophoblast binucleates may play an important role in this regulation [3,12,14-16,19]. In the current study, TIMP-2 gene expression increased in late gestation. It is difficult to clarify whether TIMP-2 plays a role in the inhibition of MMP-2 or a stimulatory function as a cytokine during this period. Although a microarray analysis supplies comprehensive information, the complex regulatory mechanisms that exist for matrix degradation and remodeling remain unclear. A possible way to examine the complex regulation of MMP is comparative studies in different species with microarrays, with the aim of finding various common genes profiles.

## Conclusion

Gelatinase activity is an important factor during implantation in cows as well as in various species that have an invasive type placenta like humans and rodents. MMP-2 gene and protein expression during peri-implantation coincides with ECM degradation in cows. Quantitative PCR and microarray analysis suggest that MMP-2 also participates as a regulatory factor for placental release during labor, and its function may be adjusted by other MMP related molecules, stimulators, and inhibitors.

## Competing interests

The authors declare that they have no competing interests.

## Authors' contributions

KK and KU are both co-first authors and contributed equally to this study. They participated in the design of the study, carried out most of the experiments, and wrote the manuscript. OY carried out the *in situ *zymography. TT, TS, and AI participated in coordinating the design of the study. JT supplied tissue samples. KH planned and participated in coordinating the design of the study, contributed to drafting the manuscript, and supervised the process. All authors have read and approved the final manuscript.
